# 2-[(Cyclo­penta-1,3-dien-2-yl)diphenyl­meth­yl]-1-methyl-1*H*-imidazole

**DOI:** 10.1107/S1600536809055561

**Published:** 2010-01-09

**Authors:** Qi Sun, Wanli Nie, Maxim V. Borzov

**Affiliations:** a Key Laboratory of Synthetic and Natural Functional Molecular Chemistry of the Ministry of Education, College of Chemistry and Materials Science, The North-West University of Xi’an, Tai Bai Bei avenue 229, Xi’an 710069, Shaanxi Province, People’s Republic of China

## Abstract

The title compound, C_22_H_20_N_2_, (I*b*), forms along with 2-[(cyclo­penta-1,3-dien-1-yl)diphenyl­meth­yl]-1-methyl-1*H*-imid­azole, (I*a*), which differs with respect to the position of the double-bonds in the C_5_H_5_ ring,  in an approximately 3:7 ratio (I*a*:I*b*; NMR spectroscopy data). However, in a single crystal, only compound (I*b*) is present. H atoms of the CH_2_ group (C_5_H_5_ ring) were found from the difference Fourier synthesis and refined isotropically using the riding model. Hypothesis on possible presence of the (I*a*) isomer in crystal lattice (model with a C_5_H_5_ ring disordered between two positions) was especially checked and rejected due to its inconsistency. In the crystal structure, no significant hydrogen-bonding inter­actions between the CH_2_ groups of the C_5_H_5_ rings and nonsubstituted N-atoms of the imidazole rings were observed. Despite the fact that the chemically achiral compound (I) crystallizes in a chiral space group *P*2_1_2_1_2_1_, neither the absolute structure determination nor assignment of the inversion twinning was possible in the absence of a heavy atom.

## Related literature

For the structural parameters of mono-alkyl substituted cyclo­penta­dienes (organic structures only), see: Tacke *et al.* (2001 ▶); Liebling & Marsh (1965 ▶); Haumann *et al.*(1996 ▶); Deck *et al.* (2004 ▶); Malpass *et al.* (2004 ▶); Cheung *et al.* (2005 ▶); Bauer *et al.* (2001 ▶); Huerlander *et al.* (2002 ▶); Millelr & Bercaw (2004 ▶); Li *et al.* (2005 ▶); Brunner *et al.* (2004 ▶); Otero *et al.* (2007 ▶); Hutton *et al.* (2008 ▶). For the structural parameters of 1,2-dialkyl-1*H*-imidazoles (organic structures only, not bi- or oligocyclic, non-ionic), see: Bruijnincx *et al.* (2005 ▶); Aakeroy *et al.* (2006 ▶); Zhang *et al.* (2007 ▶); Upadhyaya *et al.* (1997 ▶); Braussaud *et al.* (2001 ▶); Peters *et al.* (2005 ▶); Laus *et al.* (2008 ▶). For the structural parameters of Li, Ti, and Zr complexes derived from 1*H*-imidazol(in)-2-yl side-chain-functionalized cyclo­penta­dienes, see: Krut’ko *et al.* (2006 ▶); Nie *et al.* (2008 ▶); Wang *et al.* (2009 ▶). For a description of the Cambridge Structural database, see: Allen (2002 ▶).
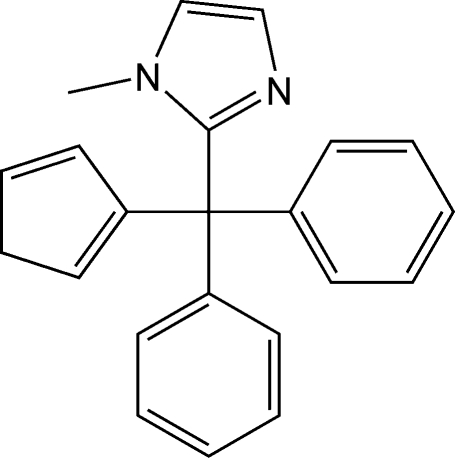


## Experimental

### 

#### Crystal data

C_22_H_20_N_2_
*M* *_r_* = 312.40
Orthorhombic, 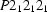

*a* = 10.563 (5) Å
*b* = 10.603 (5) Å
*c* = 15.185 (7) Å
*V* = 1700.6 (14) Å^3^
*Z* = 4
Mo *K*α radiation
μ = 0.07 mm^−1^
*T* = 295 K
0.20 × 0.05 × 0.05 mm

#### Data collection

Bruker SMART APEXII diffractometer
Absorption correction: multi-scan (*SADABS*; Sheldrick, 1996 ▶) *T*_min_ = 0.986, *T*_max_ = 0.996
8785 measured reflections
1922 independent reflections
1543 reflections with *I* > 2σ(*I*)
*R*_int_ = 0.033

#### Refinement

*R*[*F* ^2^ > 2σ(*F* ^2^)] = 0.040
*wR*(*F* ^2^) = 0.120
*S* = 1.04
1922 reflections
218 parameters
5 restraints
H-atom parameters constrained
Δρ_max_ = 0.27 e Å^−3^
Δρ_min_ = −0.11 e Å^−3^

### 

Data collection: *APEX2* (Bruker, 2007 ▶); cell refinement: *SAINT* (Bruker, 2007 ▶); data reduction: *SAINT*; program(s) used to solve structure: *SHELXS97* (Sheldrick, 2008 ▶); program(s) used to refine structure: *SHELXL97* (Sheldrick, 2008 ▶); molecular graphics: *SHELXTL* (Sheldrick, 2008 ▶) and *OLEX2* (Dolomanov *et al.*, 2009 ▶); software used to prepare material for publication: *SHELXTL* and *OLEX2*.

## Supplementary Material

Crystal structure: contains datablocks I, global. DOI: 10.1107/S1600536809055561/dn2523sup1.cif

Structure factors: contains datablocks I. DOI: 10.1107/S1600536809055561/dn2523Isup2.hkl

Additional supplementary materials:  crystallographic information; 3D view; checkCIF report

## Figures and Tables

**Fig. 1.**
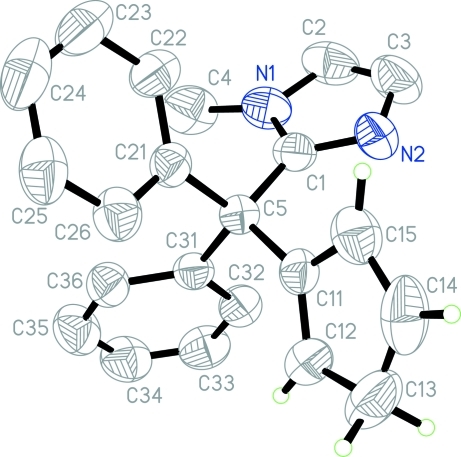
A view of the molecule of compound (I). Displacement ellipsoids are shown at the 50% probability level. All H-atoms except of those in the C5H5-ring are omitted for clarity.

**Fig. 2.**
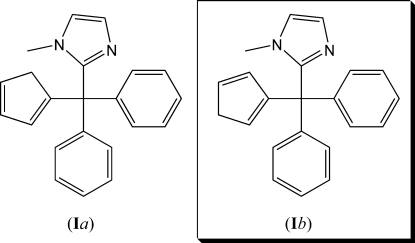
Isomers (Ia) and (Ib).

## supplementary crystallographic information

### Comment

1*H*-Imidazol(in)-2-yl side-chain functionalized cyclopentadienes were introduced as ligands into the organometallic chemistry, and, particularly into that of the Group 4 transition metals, not long ago (Krut'ko *et al.*, 2006; Nie *et al.*, 2008; Wang *et al.*, 2009). This contribution reports the first structural characterization of a ligand of question in its CH-acid form.

The title compound, C_22_H_20_N_2_, (I), presents a mixture of two isomers on the substituent position in respect to the double bond system in the C_5_H_5_-ring, 2-[(cyclopenta-1,3-dien-1-yl]diphenylmethyl]-1-methyl-1*H*-imidazole, (I*a*), and 2-[(cyclopenta-1,3-dien-2-yl]diphenylmethyl]-1-methyl-1*H*-imidazole, (I*b*), what are formed in an approximately 3: 7 ratio. However, in a single-crystal, only compound (I*b*) is present. H-atoms of the CH_2_-group (C_5_H_5_-ring) were found from the difference Fourier synthesis and refined isotropically using the riding model. Hypothesis on possible presence of the (I*a*) isomer in crystal lattice (model with a C_5_H_5_-ring disordered between two positions) was especially checked and rejected due to its inconsistency. The Cp-ring is planar within 0.004 Å, with the bridging carbon C5 deviating from the r.m.s. C11 through C15 plane by 0.084 (4) Å. As for the rest of the molecule, all the bond lengths and angles are within normal ranges (see Related literature section). Both phenyl rings C21 through C26 and C31 through C36 are planar within 0.02 Å. The imidazole moiety C1/N1/C2/C3/N2/ is planar within 0.005 Å, with atoms C4 (1-methyl group) and C5 (bridging carbon atom) deviating from the imidazole ring plane by 0.091 (5) and 0.021 (4) Å, respectively. No special intermolecular contacts in the crystal lattice were observed. Despite the fact that a chemically achiral compound (I) crystallizes in a chiral space group *P*2_1_2_1_2_1_, neither the absolute structure determination nor approval of the inversion twinning was possible due to evident reasons (Mo-*K*α radiation with no atoms heavier than nitrogen).

### Experimental

All operations were performed on an Ar-vacuum line using the conventional Schlenk technique. — NMR spectra were recorded on Varian INOVA-400 instrument in CDCl_3_ at 298 K. For ^1^H and ^13^C{^1^H} spectra, the TMS resonances (δ_H_ = 0.0 and δ_C_ = 0.0) were used as internal reference standards. — Chromato-mass spectrum was measured on Agilent 6890 Series GC system equipped with HP 5973 mass-selective detector. — The elemental analysis was performed on the Vario ELIII CHNOS automated analyzer.

To a solution of 1-methyl-1*H*-imidazole (1.23 g, 15.0 mmol) in tetrahydrofuran (THF; 60 ml), a solution of *n*-BuLi in hexane (8.3 ml, 1.82 *M*, 15.1 mmol) was added under stirring at 195 K (acetone bath) during 30 min. The reaction mixture was stirred at the same temperature for additional 45 min, that gave a bright-yellow solution. To this slurry, a solution of 6,6-diphenylfulvene (3.45 g, 15.0 mmol) in THF (45 ml) was added dropwise during 30 min. The color of the reaction mixture turned brown. The reaction mixture was allowed to warm up gradually up to ambient temperature and the stirring was continued for the next 12 h. The mixture was quenched with water (50 ml; ice-bath cooling), the organic phase was separated, the water phase was extracted with CH_2_Cl_2_ (3 × 30 ml), and the combined extracts were dried with Na_2_SO_4_. Concentrating of the organic extracts on a rotary evaporator gave crude (I) as a brown solid. Crude (I) was refluxed with Et_2_O (10 ml) cooled down to room temperature and the filtered-off solid was recrystallized from CH_2_Cl_2_ – Et_2_O mixture (1.8: 10) that gave pure (I) as yellowish crystals. Yield 1.6 g, (34%). — ^1^H NMR: δ = 2.92 (s, 3 H, NCH_3_, *a*), 2.96 (s, 3 H, NCH_3_, *b*), 3.05 (m, 2 H, CH_2_ in Cp, *b*), 3.10 (m, 2 H, CH_2_ in Cp, *a*), 5.91, 6.05, 6.27, 6.28, 6.37, 6.38 (all m, all 1 H in respective ratios, CH in Cp, *a*,*b*), 6.84, 7.07 (both d, 1 H + 1 H, ^3^*J*_HH_ = 1.22 Hz, CH=CH in imidazole, *b*), 6.85, 7.06 (both d, 1 H + 1 H, ^3^*J*_HH_ = 1.22 Hz, CH=CH in imidazole, *a*), 7.21–7.32 (m, 10 H in respect to the sum of the relative integral intensities for *a* and *b*, CH in Ph, *a*,*b*). Molar ratio *a*: *b *equals to 3: 7. — ^13^C{^1^H} NMR: δ = 34.75 (NCH_3_, *b*), 34.90 (NCH_3_, *a*), 40.32 (CH_2_ in Cp, *b*), 43.85 (CH_2_ in Cp, *a*), 57.14 (CPh_2_, *b*), 58.02 (CPh_2_, *a*), 122.31, 126.63 (CH=CH in imidazole, *a*,*b*), 126.43 (*p*-CH in Ph, *a*), 126.51 (*p*-CH in Ph, *b*), 127.73, 129.38 (*o*- and *m*-CH in Ph, *b*), 127.79, 129.16 (*o*- and *m*-CH in Ph, *a*), 128.76, 131.98, 135.94 (CH in Cp, *b*), 130.58, 130.91, 134.00 (CH in Cp, *a*), 142.67 (*ipso*-C in Ph, *b*), 143.32 (*ipso*-C in Ph, *a*), 150.14 (N—C=N, *b*), 150.67 (N—C=N, *a*), 150.60 (C in Cp, *b*), 153.13 (C in Cp, *a*). — EI MS (70 eV) m/*z* (%): 312 (100) [*M*], 311 (66) [*M* – H], 297 (3) [*M* – CH_3_], 285 (34) [*M* – HCN], 247 (9) [*M* – C_5_H_5_], 235 (72) [*M* – Ph].— Anal. Found: C, 83.81; H, 6.35; N, 8.76%. C_22_H_20_N_2_ Calc.: C, 84.58; H, 6.45; N, 8.97%. Single crystal of (I) suitable for X-ray diffraction analysis was obtained by crystallization of (I) from CH_2_Cl_2_ – Et_2_O mixture (1: 6 vol.)

### Refinement

Non-H atoms were refined anisotropically. H atoms were treated as riding atoms with distances C—H = 0.96 (CH_3_), 0.97 (CH_2_), 0.93 Å (C_Ar_H), and *U*_iso_(H) = 1.5 *U*_eq_(C), 1.2 *U*_eq_(C), and 1.2 *U*_eq_(C), respectively. The components of the anisotropic displacement parameters (ADP-s) for C12 through C15-atoms of the C_5_H_4_-group along 1,2- and 1,3-directions were restrained to be the same with su of 0.002 Å^2^ (DELU instruction). Despite the fact that an achiral compound (I) crystallizes in a chiral space group *P*2_1_2_1_2_1_, neither the absolute structure determination nor approval of the inversion twinning was possible due to evident reasons (Mo-*K*α radiation with no atoms heavier than nitrogen). Thus, the Friedel opposites were merged and treated as equivalents.

### Crystal data

**Table:**

C_22_H_20_N_2_	*F*(000) = 664
*M**_r_* = 312.40	*D*_x_ = 1.220 Mg m^−^^3^
Orthorhombic, *P*2_1_2_1_2_1_	Mo *K*α radiation, λ = 0.71073 Å
Hall symbol: P 2ac 2ab	Cell parameters from 5639 reflections
*a* = 10.563 (5) Å	θ = 2.3–28.0°
*b* = 10.603 (5) Å	µ = 0.07 mm^−^^1^
*c* = 15.185 (7) Å	*T* = 295 K
*V* = 1700.6 (14) Å^3^	Prism, colorless
*Z* = 4	0.20 × 0.05 × 0.05 mm

### Data collection

**Table:**

Bruker SMART APEXII diffractometer	1922 independent reflections
Radiation source: fine-focus sealed tube	1543 reflections with *I* > 2σ(*I*)
graphite	*R*_int_ = 0.033
Detector resolution: 8.333 pixels mm^-1^	θ_max_ = 26.0°, θ_min_ = 2.3°
phi and ω scans	*h* = −12→13
Absorption correction: multi-scan (*SADABS*; Sheldrick, 1996)	*k* = −13→13
*T*_min_ = 0.986, *T*_max_ = 0.996	*l* = −10→18
8785 measured reflections	

### Refinement

**Table:**

Refinement on *F*^2^	Primary atom site location: structure-invariant direct methods
Least-squares matrix: full	Secondary atom site location: difference Fourier map
*R*[*F*^2^ > 2σ(*F*^2^)] = 0.040	Hydrogen site location: inferred from neighbouring sites
*wR*(*F*^2^) = 0.120	H-atom parameters constrained
*S* = 1.03	*w* = 1/[σ^2^(*F*_o_^2^) + (0.0736*P*)^2^ + 0.110*P*] where *P* = (*F*_o_^2^ + 2*F*_c_^2^)/3
1922 reflections	(Δ/σ)_max_ < 0.001
218 parameters	Δρ_max_ = 0.27 e Å^−^^3^
5 restraints	Δρ_min_ = −0.11 e Å^−^^3^

### Special details

**Table:**

Geometry. All e.s.d.'s (except the e.s.d. in the dihedral angle between two l.s. planes) are estimated using the full covariance matrix. The cell e.s.d.'s are taken into account individually in the estimation of e.s.d.'s in distances, angles and torsion angles; correlations between e.s.d.'s in cell parameters are only used when they are defined by crystal symmetry. An approximate (isotropic) treatment of cell e.s.d.'s is used for estimating e.s.d.'s involving l.s. planes.
Refinement. Refinement of *F*^2^ against ALL reflections. The weighted *R*-factor *wR* and goodness of fit *S* are based on *F*^2^, conventional *R*-factors *R* are based on *F*, with *F* set to zero for negative *F*^2^. The threshold expression of *F*^2^ > σ(*F*^2^) is used only for calculating *R*-factors(gt) *etc*. and is not relevant to the choice of reflections for refinement. *R*-factors based on *F*^2^ are statistically about twice as large as those based on *F*, and *R*- factors based on ALL data will be even larger.

### Fractional atomic coordinates and isotropic or equivalent isotropic displacement parameters (Å^2^)

**Table:**

	*x*	*y*	*z*	*U*_iso_*/*U*_eq_	
N1	0.1464 (2)	0.6352 (2)	0.49551 (13)	0.0578 (6)	
N2	0.2989 (2)	0.73895 (18)	0.56444 (14)	0.0588 (6)	
C1	0.2381 (2)	0.6304 (2)	0.55926 (15)	0.0460 (5)	
C2	0.1512 (3)	0.7552 (3)	0.4606 (2)	0.0725 (8)	
H2	0.0996	0.7876	0.4164	0.087*	
C3	0.2442 (3)	0.8160 (3)	0.5025 (2)	0.0756 (9)	
H3	0.2685	0.8987	0.4914	0.091*	
C4	0.0623 (3)	0.5371 (3)	0.4627 (2)	0.0785 (9)	
H4C	0.0274	0.4912	0.5115	0.118*	
H4A	0.1091	0.4805	0.4256	0.118*	
H4B	−0.0050	0.5749	0.4294	0.118*	
C5	0.2651 (2)	0.5151 (2)	0.61653 (14)	0.0412 (5)	
C11	0.3890 (2)	0.5365 (2)	0.66661 (15)	0.0457 (5)	
C12	0.4947 (2)	0.4661 (3)	0.66347 (17)	0.0561 (6)	
H12	0.5063	0.3969	0.6267	0.067*	
C13	0.5894 (3)	0.5134 (3)	0.7259 (2)	0.0778 (8)	
H13A	0.6656	0.5395	0.6954	0.093*	
H13B	0.6113	0.4489	0.7686	0.093*	
C14	0.5283 (3)	0.6217 (3)	0.76939 (19)	0.0790 (8)	
H14	0.5641	0.6719	0.8130	0.095*	
C15	0.4055 (3)	0.6363 (2)	0.73309 (18)	0.0616 (6)	
H15	0.3464	0.6974	0.7484	0.074*	
C21	0.1592 (2)	0.4951 (2)	0.68599 (14)	0.0443 (5)	
C22	0.0450 (2)	0.5593 (3)	0.68353 (18)	0.0580 (7)	
H22	0.0312	0.6208	0.6409	0.070*	
C23	−0.0488 (3)	0.5321 (3)	0.7443 (2)	0.0754 (9)	
H23	−0.1253	0.5754	0.7420	0.090*	
C24	−0.0307 (3)	0.4438 (3)	0.8067 (2)	0.0807 (10)	
H24	−0.0959	0.4237	0.8453	0.097*	
C25	0.0844 (3)	0.3828 (3)	0.81354 (18)	0.0688 (8)	
H25	0.0980	0.3246	0.8584	0.083*	
C26	0.1786 (3)	0.4083 (2)	0.75399 (16)	0.0562 (6)	
H26	0.2562	0.3675	0.7589	0.067*	
C31	0.2772 (2)	0.3977 (2)	0.55630 (14)	0.0430 (5)	
C32	0.3568 (2)	0.4048 (2)	0.48296 (16)	0.0537 (6)	
H32	0.4036	0.4777	0.4728	0.064*	
C33	0.3665 (3)	0.3046 (3)	0.42553 (18)	0.0650 (7)	
H33	0.4188	0.3112	0.3765	0.078*	
C34	0.3004 (3)	0.1959 (3)	0.4395 (2)	0.0713 (8)	
H34	0.3082	0.1288	0.4004	0.086*	
C35	0.2234 (3)	0.1862 (3)	0.5106 (2)	0.0707 (8)	
H35	0.1785	0.1120	0.5204	0.085*	
C36	0.2110 (2)	0.2860 (2)	0.56874 (17)	0.0536 (6)	
H36	0.1575	0.2780	0.6170	0.064*	

### Atomic displacement parameters (Å^2^)

**Table:**

	*U*^11^	*U*^22^	*U*^33^	*U*^12^	*U*^13^	*U*^23^
N1	0.0615 (14)	0.0586 (12)	0.0533 (11)	0.0116 (11)	−0.0076 (11)	0.0052 (11)
N2	0.0645 (14)	0.0426 (10)	0.0692 (13)	−0.0012 (10)	0.0033 (12)	0.0092 (10)
C1	0.0457 (13)	0.0445 (12)	0.0479 (11)	0.0063 (11)	0.0039 (10)	0.0027 (10)
C2	0.085 (2)	0.0667 (17)	0.0661 (16)	0.0255 (17)	−0.0024 (17)	0.0166 (16)
C3	0.090 (2)	0.0497 (14)	0.087 (2)	0.0101 (16)	0.0023 (19)	0.0205 (15)
C4	0.073 (2)	0.082 (2)	0.0800 (19)	0.0081 (17)	−0.0301 (17)	−0.0082 (18)
C5	0.0389 (11)	0.0402 (11)	0.0445 (11)	0.0008 (10)	0.0002 (9)	0.0015 (9)
C11	0.0440 (12)	0.0439 (12)	0.0491 (12)	−0.0060 (10)	0.0033 (10)	0.0054 (11)
C12	0.0452 (13)	0.0594 (14)	0.0638 (14)	0.0000 (12)	−0.0020 (11)	−0.0009 (12)
C13	0.0457 (13)	0.103 (2)	0.0843 (19)	−0.0100 (13)	−0.0138 (13)	0.0156 (17)
C14	0.0928 (19)	0.0814 (18)	0.0630 (16)	−0.0413 (15)	−0.0177 (15)	0.0016 (14)
C15	0.0736 (15)	0.0530 (13)	0.0584 (14)	−0.0067 (13)	0.0016 (13)	−0.0091 (14)
C21	0.0438 (12)	0.0425 (11)	0.0467 (11)	−0.0046 (10)	0.0029 (10)	−0.0078 (11)
C22	0.0454 (13)	0.0666 (16)	0.0621 (15)	0.0010 (12)	0.0014 (12)	−0.0066 (15)
C23	0.0467 (14)	0.095 (2)	0.085 (2)	−0.0048 (15)	0.0135 (15)	−0.017 (2)
C24	0.073 (2)	0.092 (2)	0.077 (2)	−0.0333 (19)	0.0245 (18)	−0.0198 (19)
C25	0.083 (2)	0.0648 (17)	0.0583 (15)	−0.0207 (16)	0.0146 (15)	0.0004 (14)
C26	0.0612 (15)	0.0536 (13)	0.0539 (13)	−0.0056 (12)	0.0048 (12)	−0.0029 (12)
C31	0.0394 (11)	0.0423 (11)	0.0474 (11)	0.0039 (10)	−0.0033 (10)	−0.0012 (9)
C32	0.0508 (14)	0.0553 (13)	0.0552 (13)	0.0013 (12)	0.0043 (12)	−0.0002 (12)
C33	0.0619 (16)	0.0782 (18)	0.0550 (14)	0.0102 (15)	0.0035 (13)	−0.0121 (15)
C34	0.0768 (19)	0.0639 (17)	0.0734 (18)	0.0084 (16)	−0.0021 (16)	−0.0177 (16)
C35	0.081 (2)	0.0531 (14)	0.0779 (18)	−0.0053 (15)	−0.0032 (18)	−0.0113 (14)
C36	0.0538 (14)	0.0488 (13)	0.0583 (14)	−0.0027 (11)	0.0019 (13)	−0.0059 (11)

### Geometric parameters (Å, °)

**Table:**

N1—C1	1.370 (3)	C15—H15	0.9300
N1—C2	1.380 (3)	C21—C22	1.386 (3)
N1—C4	1.456 (4)	C21—C26	1.398 (3)
N2—C1	1.321 (3)	C22—C23	1.384 (4)
N2—C3	1.373 (3)	C22—H22	0.9300
C1—C5	1.527 (3)	C23—C24	1.346 (5)
C2—C3	1.336 (4)	C23—H23	0.9300
C2—H2	0.9300	C24—C25	1.381 (5)
C3—H3	0.9300	C24—H24	0.9300
C4—H4C	0.9600	C25—C26	1.372 (4)
C4—H4A	0.9600	C25—H25	0.9300
C4—H4B	0.9600	C26—H26	0.9300
C5—C11	1.530 (3)	C31—C36	1.388 (3)
C5—C31	1.550 (3)	C31—C32	1.398 (3)
C5—C21	1.552 (3)	C32—C33	1.378 (3)
C11—C12	1.344 (3)	C32—H32	0.9300
C11—C15	1.473 (3)	C33—C34	1.364 (4)
C12—C13	1.466 (4)	C33—H33	0.9300
C12—H12	0.9300	C34—C35	1.357 (4)
C13—C14	1.474 (5)	C34—H34	0.9300
C13—H13A	0.9700	C35—C36	1.384 (4)
C13—H13B	0.9700	C35—H35	0.9300
C14—C15	1.417 (4)	C36—H36	0.9300
C14—H14	0.9300		
			
C1—N1—C2	106.3 (2)	C14—C15—C11	107.3 (3)
C1—N1—C4	130.2 (2)	C14—C15—H15	126.4
C2—N1—C4	123.4 (2)	C11—C15—H15	126.4
C1—N2—C3	105.8 (2)	C22—C21—C26	118.1 (2)
N2—C1—N1	110.7 (2)	C22—C21—C5	122.9 (2)
N2—C1—C5	124.9 (2)	C26—C21—C5	119.1 (2)
N1—C1—C5	124.4 (2)	C23—C22—C21	120.2 (3)
C3—C2—N1	106.8 (3)	C23—C22—H22	119.9
C3—C2—H2	126.6	C21—C22—H22	119.9
N1—C2—H2	126.6	C24—C23—C22	120.8 (3)
C2—C3—N2	110.5 (3)	C24—C23—H23	119.6
C2—C3—H3	124.8	C22—C23—H23	119.6
N2—C3—H3	124.8	C23—C24—C25	120.3 (3)
N1—C4—H4C	109.5	C23—C24—H24	119.8
N1—C4—H4A	109.5	C25—C24—H24	119.8
H4C—C4—H4A	109.5	C26—C25—C24	119.8 (3)
N1—C4—H4B	109.5	C26—C25—H25	120.1
H4C—C4—H4B	109.5	C24—C25—H25	120.1
H4A—C4—H4B	109.5	C25—C26—C21	120.7 (3)
C1—C5—C11	108.92 (18)	C25—C26—H26	119.7
C1—C5—C31	108.80 (16)	C21—C26—H26	119.7
C11—C5—C31	110.03 (17)	C36—C31—C32	117.2 (2)
C1—C5—C21	111.18 (18)	C36—C31—C5	124.3 (2)
C11—C5—C21	107.37 (17)	C32—C31—C5	118.42 (19)
C31—C5—C21	110.53 (17)	C33—C32—C31	120.4 (2)
C12—C11—C15	108.9 (2)	C33—C32—H32	119.8
C12—C11—C5	127.6 (2)	C31—C32—H32	119.8
C15—C11—C5	123.3 (2)	C34—C33—C32	121.0 (3)
C11—C12—C13	110.7 (3)	C34—C33—H33	119.5
C11—C12—H12	124.6	C32—C33—H33	119.5
C13—C12—H12	124.6	C35—C34—C33	119.7 (3)
C12—C13—C14	104.9 (2)	C35—C34—H34	120.2
C12—C13—H13A	110.8	C33—C34—H34	120.2
C14—C13—H13A	110.8	C34—C35—C36	120.4 (3)
C12—C13—H13B	110.8	C34—C35—H35	119.8
C14—C13—H13B	110.8	C36—C35—H35	119.8
H13A—C13—H13B	108.8	C35—C36—C31	121.2 (2)
C15—C14—C13	108.1 (2)	C35—C36—H36	119.4
C15—C14—H14	125.9	C31—C36—H36	119.4
C13—C14—H14	125.9		
			
C3—N2—C1—N1	0.3 (3)	C1—C5—C21—C22	11.7 (3)
C3—N2—C1—C5	−179.4 (2)	C11—C5—C21—C22	130.7 (2)
C2—N1—C1—N2	−0.8 (3)	C31—C5—C21—C22	−109.2 (2)
C4—N1—C1—N2	175.5 (3)	C1—C5—C21—C26	−168.7 (2)
C2—N1—C1—C5	178.9 (2)	C11—C5—C21—C26	−49.7 (2)
C4—N1—C1—C5	−4.8 (4)	C31—C5—C21—C26	70.3 (2)
C1—N1—C2—C3	0.9 (3)	C26—C21—C22—C23	−3.4 (4)
C4—N1—C2—C3	−175.7 (3)	C5—C21—C22—C23	176.2 (2)
N1—C2—C3—N2	−0.7 (3)	C21—C22—C23—C24	0.2 (4)
C1—N2—C3—C2	0.2 (3)	C22—C23—C24—C25	3.0 (5)
N2—C1—C5—C11	−11.2 (3)	C23—C24—C25—C26	−3.0 (4)
N1—C1—C5—C11	169.1 (2)	C24—C25—C26—C21	−0.3 (4)
N2—C1—C5—C31	−131.2 (2)	C22—C21—C26—C25	3.5 (3)
N1—C1—C5—C31	49.2 (3)	C5—C21—C26—C25	−176.1 (2)
N2—C1—C5—C21	106.9 (3)	C1—C5—C31—C36	−128.3 (2)
N1—C1—C5—C21	−72.8 (2)	C11—C5—C31—C36	112.4 (2)
C1—C5—C11—C12	−120.6 (3)	C21—C5—C31—C36	−6.0 (3)
C31—C5—C11—C12	−1.5 (3)	C1—C5—C31—C32	49.8 (3)
C21—C5—C11—C12	118.9 (3)	C11—C5—C31—C32	−69.5 (2)
C1—C5—C11—C15	64.5 (3)	C21—C5—C31—C32	172.10 (19)
C31—C5—C11—C15	−176.32 (19)	C36—C31—C32—C33	0.9 (3)
C21—C5—C11—C15	−56.0 (3)	C5—C31—C32—C33	−177.3 (2)
C15—C11—C12—C13	−0.6 (3)	C31—C32—C33—C34	−1.0 (4)
C5—C11—C12—C13	−176.1 (2)	C32—C33—C34—C35	0.4 (5)
C11—C12—C13—C14	0.7 (3)	C33—C34—C35—C36	0.3 (5)
C12—C13—C14—C15	−0.5 (3)	C34—C35—C36—C31	−0.4 (4)
C13—C14—C15—C11	0.1 (3)	C32—C31—C36—C35	−0.2 (4)
C12—C11—C15—C14	0.3 (3)	C5—C31—C36—C35	177.9 (2)
C5—C11—C15—C14	176.0 (2)		

## References

[bb1] Aakeroy, C. B., Salmon, D. J., Smith, M. M. & Desper, J. (2006). *Cryst. Growth Des.* **6**, 1033–1042.

[bb2] Allen, F. H. (2002). *Acta Cryst.* B**58**, 380–388. 10.1107/s0108768102003890 12037359

[bb3] Bauer, A., Hilbig, H., Hiller, W., Hinterschwepfinger, E., Kohler, F. H. & Neumayer, M. (2001). *Synthesis*, pp. 778–782.

[bb4] Braussaud, N., Ruther, T., Kavell, K. J., Skelton, B. W. & White, A. H. (2001). *Synthesis*, pp. 626–632.

[bb5] Bruijnincx, P. C. A., Lutz, M., Spek, A. L., van Faassen, E. E., Weckhuysen, B. M., van Koten, G. & Gebbink, R. J. M. K. (2005). *Eur. J. Inorg. Chem.* pp. 779–781.

[bb6] Bruker (2007). *APEX2* and *SAINT* Bruker AXS Inc., Madison, Wisconsin, USA.

[bb7] Brunner, H., Kollnberger, A., Mehmood, A., Tsuno, T. & Zabel, M. (2004). *J. Organomet. Chem.* **689**, 4244–4262.

[bb8] Cheung, M., Chan, H. & Xie, Z. (2005). *Dalton Trans.* pp. 2375–2381. 10.1039/b504076k 15995745

[bb9] Deck, P. A., Konate, M. M., Kelly, B. V. & Slebodnik, C. (2004). *Organometallics*, **23**, 1089–1097.

[bb10] Dolomanov, O. V., Bourhis, L. J., Gildea, R. J., Howard, J. A. K. & Puschmann, H. (2009). *J. Appl. Cryst.* **42**, 339–341.

[bb11] Haumann, T., Benet-Buchholz, J. & Boese, R. (1996). *J. Mol. Struct.* **374**, 299–304.

[bb12] Huerlander, D., Frohlich, R. & Erker, G. (2002). *J. Chem. Soc. Dalton Trans.* pp. 1513–1520.

[bb13] Hutton, B. W., Macintosh, F., Ellis, D., Herisse, F., Macgregor, S. A., McKey, D., Petrie-Armstrong, V., Rosair, G. M., Perekalin, D. S., Tricas, H. & Welch, A. J. (2008). *Chem. Commun.* pp. 5345–5347. 10.1039/b810702e 18985205

[bb14] Krut’ko, D. P., Borzov, M. V., Liao, L., Nie, W., Churakov, A. V., Howard, J. A. K. & Lemenovskii, D. A. (2006). *Russ. Chem. Bull.* **55**, 1574–1580.

[bb15] Laus, G., Schwarzler, A., Bentivoglio, G., Hummel, M., Kahlenberg, V., Wurst, K., Kristeva, E., Schutz, J., Kopacka, H., Kreutz, C., Bonn, G., Andriyko, Y., Nauer, G. & Schottenberger, H. (2008). *Z. Naturforsch. Teil B*, **63**, 447–464.

[bb16] Li, B., Wang, B., Xu, S. & Zhou, X. (2005). *J. Organomet. Chem.* **690**, 5309–5317.

[bb17] Liebling, G. & Marsh, R. E. (1965). *Acta Cryst.* **19**, 202–205.

[bb18] Malpass, J. R., Skerry, P. S. & Rimmington, S. L. (2004). *Heterocycles*, **62**, 679–691.

[bb19] Millelr, S. A. & Bercaw, J. E. (2004). *Organometallics*, **23**, 1777–1789.

[bb20] Nie, W., Liao, L., Xu, W., Borzov, M. V., Krut’ko, D. P., Churakov, A. V., Howard, J. A. K. & Lemenovskii, D. A. (2008). *J. Organomet. Chem.* **693**, 2355–2368.

[bb21] Otero, A., Fernandez-Baeza, J., Antinolo, A., Tejeda, J., Lara-Sanchez, A., Sanchez-Barba, L. F., Sanchez-Molina, M. & Rodriguez, A. M. (2007). *Organometallics*, **26**, 4310–4320.

[bb22] Peters, L., Hubner, E. & Burzlaff, N. (2005). *J. Organomet. Chem.* **690**, 2009–2016.

[bb23] Sheldrick, G. M. (1996). *SADABS* University of Göttingen, Germany.

[bb24] Sheldrick, G. M. (2008). *Acta Cryst.* A**64**, 112–122. 10.1107/S0108767307043930 18156677

[bb25] Tacke, M., Dunne, J. P., Fox, S., Linti, G. & Teuber, R. (2001). *J. Mol. Struct.* **570**, 197–202.

[bb26] Upadhyaya, S. P., Davis, F. S., Lee, J. J., Zaw, K., Bauer, R. & Heimer, N. E. (1997). *J. Heterocycl. Chem.* **34**, 1607–1620.

[bb27] Wang, X., Nie, W., Ge, F. & Borzov, M. V. (2009). *Acta Cryst.* C**65**, m255–m259. 10.1107/S010827010902112X 19578257

[bb28] Zhang, D., Aihara, H., Watanabe, T., Matsuo, T. & Kawaguchi, H. (2007). *J. Organomet. Chem.* **692**, 234–242.

